# Revision of the genus *Hemisaprinus* Kryzhanovskij, 1976 (Coleoptera, Histeridae, Saprininae)

**DOI:** 10.3897/zookeys.429.7949

**Published:** 2014-07-30

**Authors:** Tomáš Lackner

**Affiliations:** 1 Czech University of Life Sciences, Faculty of Forestry and Wood Sciences, Department of Forest Protection and Entomology, Kamýcká 1176, CZ-165 21 Praha 6 – Suchdol, Czech Republic

**Keywords:** Coleoptera, Histeridae, Saprininae, *Hemisaprinus*, Palaearctic Region, taxonomic revision

## Abstract

The monophyletic genus *Hemisaprinus* Kryzhanovskij in Kryzhanovskij & Reichardt, 1976 is revised herein. All three species *Hemisaprinus subvirescens* (Ménétries, 1832), *H. lutshniki* (Reichardt, 1941) and *H. cyprius* (Dahlgren, 1981) are found to be correctly assigned to the genus and their monophyly is supported by the synapomorphy of the presence of prosternal foveae. The three species are re-described and supplemented with colour photographs as well as SEM micrographs outlining their differences. Male genitalia drawing of *H. subvirescens* and *H. lutshniki* are provided and a key to the species is given. *Hemisaprinus subvirescens* (Ménétries, 1832) is newly reported from Armenia, Azerbaijan, Kyrgyzstan, Uzbekistan, Turkmenistan, Tajikistan, Jordan, Cyprus and Mongolia. The lectotypes and paralectotypes of the following species are designated herein: *Saprinus foveisternus* Schmidt, 1884, *Saprinus syriacus* Marseul, 1855 and *Saprinus viridulus* Marseul, 1855.

## Introduction

The genus *Hemisaprinus* was established by Kryzhanovskij in Kryzhanovskij and Reichardt (1976) based on the species *Hister subvirescens* Ménétries, 1832. At the time of its designation *Hemisaprinus* was a mere subgenus of the genus *Saprinus* Erichson, 1834 and Kryzhanovskij and Reichardt (1976) included in it another species, *Saprinus lutshniki* Reichardt, 1941 which was until then treated only as an aberration of *Saprinus cribellatus* Marseul, 1855. Kryzhanovskij and Reichardt (1976) used the presence of the prosternal foveae as the discriminating character for the erection of the new subgenus. *Hemisaprinus* remained as a subgenus of *Saprinus* until Mazur (2011) elevated its rank to a fully-fledged genus without any explanation or justification for his action. Dahlgren (1981) described *Saprinus cyprius* from Cyprus, remarking that this species should probably not be included in the subgenus *Hemisaprinus*, since it is morphologically different from the other two species. *Saprinus cyprius* was, however, placed into the subgenus *Hemisaprinus* by Mazur (1984, 1997, 2004, 2011). In this paper, the taxonomic status of *Hemisaprinus* as self-standing genus as well as the placement of *Saprinus cyprius* into *Hemisaprinus* are upheld and clarified in the discussion. This work presents another contribution to the on-going revisionary work of the genera of the subfamily Saprininae (Lackner 2009a, Lackner 2009b, Lackner 2009c, Lackner 2010, Lackner 2011a, Lackner 2011b; Tishechkin and Lackner 2012; Lackner 2012; Lackner 2013a, Lackner 2013b; Lackner and Gomy 2013; Lackner 2014; Lackner and Tishechkin 2014).

## Material and methods

All dry-mounted specimens were relaxed in warm water for several hours or overnight, depending on the body size. After removal from original cards, the beetles were side-mounted on triangular points and observed under a Nikon 102 stereoscopic microscope with diffused light. Body structures were studied using methods described by Ôhara (1994): male genitalia were macerated in a hot 10% KOH solution for about 15 minutes, cleared in 80% alcohol, macerated in lactic acid with fuchsine, incubated at 60°C for two hours, and subsequently transferred into a 1:1 mixture of glacial acetic acid and methyl salicylate, heated at 60°C for 15 minutes and cleared in xylene. Specimens were then observed in α-terpineol in a small glass dish. Digital photographs of the male terminalia were taken by a Nikon 4500 Coolpix camera and edited in Adobe Photoshop CS4. Based on the photographs or direct observations, the genitalia were drawn using a light-box Hakuba klv-7000. SEM photographs were taken with a JSM 6301F microscope at the laboratory of Faculty of Agriculture, Hokkaido University, Sapporo, Japan and colour images were produced by F. Slamka (Bratislava, Slovakia). All available specimens were measured with an ocular micrometre. Beetle terminology follows that of Ôhara (1994) and Lackner (2010). Separate lines of the same label are demarcated by a slash (/). The following acronyms of museums and private collections are used throughout the text:

CAS Alexander Sokolov collection, Moscow, Russia;

CND Nicolas Dégallier collection, Paris, France;

MMBC Moravské Zemské Muzeum Brno, Czech Republic (P. Baňař);

MNHN Musém National d’Histoire Naturelle, Paris, France (A. Taghavian);

MNHUB Museum für Naturkunde, Humboldt- Universität, Berlin, Germany (B. Jaeger);

MZLU Museum of Zoology Lund, Lund, Sweden (C. Fägerström);

NCB Naturalis Biodiversity Centre, Leiden, Netherlands (B. Brugge);

TLAN Tomáš Lackner collection, temporarily housed at Naturalis Biodiversity Centre, Leiden, Netherlands;

ZIN Zoological Institute, Russian Academy of Sciences, St. Petersburg, Russia (B. Kataev).

**Abbreviations.** Abbreviations of morphological measurements follow Ôhara (1994) and are used throughout the text as follows:

APW width between anterior angles of pronotum

EL length of elytron along elytral suture

EW maximum width between outer margins of elytra

PEL length between anterior angles of pronotum and apices of elytra

PPW width between posterior angles of pronotum.

## Taxonomy

### Hemisaprinus

Taxon classificationAnimaliaColeopteraHisteridae

Kryzhanovskij, 1976

Hemisaprinus Kryzhanovskij, 1976 in Kryzhanovskij and Reichardt (1976): 111, 182 (as a subgenus of *Saprinus* Erichson, 1834). Type species: *Hister subvirescens* Ménétriés, 1832, original designation.
Hemisaprinus (as a subgenus of *Saprinus* Erichson, 1834): Mazur (1984): 62; Mazur (1997): 231; Mazur (2004): 96; Lackner (2010): 63, 205.
Hemisaprinus : Mazur (2011): 188.

#### Diagnosis.

Although *Hemisaprinus* has been recently diagnosed by Lackner (2010), the published diagnosis has to be adapted with respect to the newly examined *Hemisaprinus cyprius* as follows: dark-brown to entirely black species usually with greenish hue to bi-colored species, with bronze metallic hue and partly reddish-brown elytra. Frons wholly punctate; frontal stria widely interrupted, can be slightly prolonged onto clypeus; mandibles punctate; pronotum punctate, pronotal depressions vaguely impressed to absent; pronotal hypomeron asetose; prosternal foveae present; carinal prosternal striae stopping short of prosternal foveae (*Hister subvirescens*) or entering them (*Hemisaprinus lutshniki*, *Hemisaprinus cyprius*). Lateral prosternal striae terminating in prosternal foveae (*Hister subvirescens*) or terminating near apices of carinal prosternal striae (*Hemisaprinus lutshniki*, *Hemisaprinus cyprius*). Elytra with vaguely to well-defined glabrous to sparsely punctate ‘mirror’; dorsal elytral striae 1–4 present, reaching approximately elytral half apically; in one species (*Hemisaprinus cyprius*) 2^nd^ dorsal elytral stria missing.

#### Differential diagnosis.

By the presence of prosternal foveae *Hemisaprinus* can be readily differentiated from members of the genus *Saprinus*, which it otherwise strongly resembles, by the absence of complete frontal stria as well as general appearance. The sensory structures of the antenna (Figs 3, 15) are typically *Saprinus*-like as well, with four oval sensory areas on ventral side of the club with a corresponding vesicle situated under internal distal sensory area. The reader is referred to the Key to the genera of the Palaearctic Saprininae by the author (Lackner 2010: 60) for more information.

#### Biology.

*Hemisaprinus subvirescens* is found chiefly on carcasses in arid regions while *Hemisaprinus lutshniki* is found in decomposing vegetable matter, and has not been found on carcasses so far (Lackner 2010). The biology of *Hemisaprinus cyprius* Dahlgren, 1981 is completely unknown.

#### Distribution.

This genus includes three described species: *Hemisaprinus subvirescens* (Ménétriés, 1832) known from Georgia, southern Russia, Kazakhstan, Turkey, Syria, Israel, Iran, Iraq, Afghanistan, Pakistan, Burma and China (Mazur 2011). It is herein newly reported from Azerbaijan, Tajikistan, Kyrgyzstan, Uzbekistan, Turkmenistan, Jordan, Armenia, Cyprus and Mongolia. *Hemisaprinus lutshniki* (Reichardt, 1941) is known from southern Russia, western Siberia and Kazakhstan (Mazur 2011) and *Hemisaprinus cyprius* Dahlgren, 1981 is only known from northern Cyprus: Kyrenia (Dahlgren 1981).

### Hemisaprinus subvirescens

Taxon classificationAnimaliaColeopteraHisteridae

(Ménétriés, 1832)

**Figure 1.**
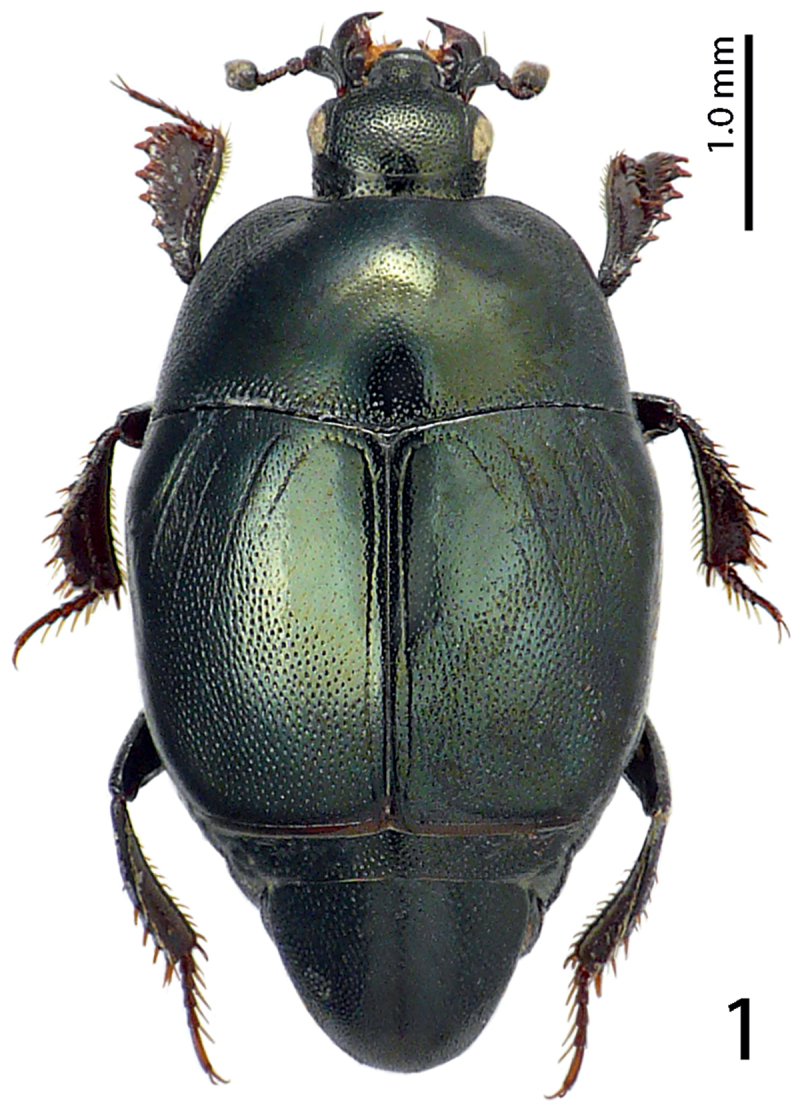
*Hemisaprinus subvirescens* (Ménétries, 1832) habitus.

Figs 1 [Fig F2] [Fig F3] [Fig F4] –12

**Figure 2. F2:**
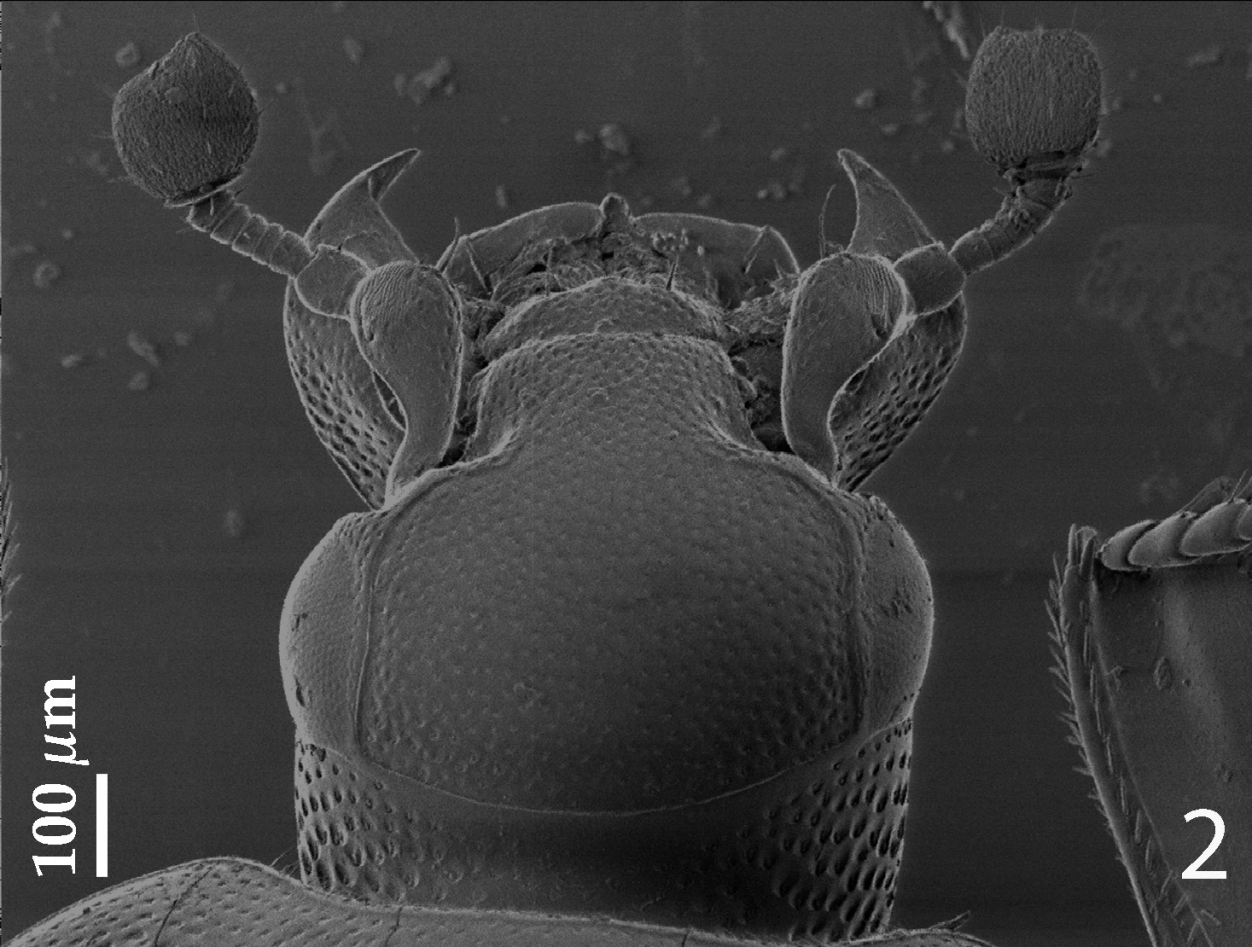
*Hemisaprinus subvirescens* (Ménétries, 1832) head, dorsal view.

Hister subvirescens Ménétriés, 1832: 171.
Hister subvirescens : Faldermann (1835): 230.
Saprinus subvirescens : Marseul (1855): 736; Reichardt (1922): 50; Reichardt (1941): 184, 240, fig. 87; Dahlgren (1968): 87, 93, figs 2G, 5A.
Saprinus (Hemisaprinus) subvirescens : Kryzhanovskij and Reichardt (1976): 127, 183, figs 357–360; Mazur (1984): 62; Mazur (1997): 231; Mazur (2004): 96; Lackner (2010): 205, figs 10, 69, 103, 135, 643–659.
Saprinus syriacus Marseul, 1855: 469. Synonymized by Reichardt (1941): 240.
Saprinus viridulus Marseul, 1855: 468. Synonymized by Dahlgren (1968): 87.
Saprinus foveisternus Schmidt, 1884: 9. Synonymized by Auzat (1920): 3.
Hemisaprinus subvirescens : Mazur (2011): 188.

#### Type locality.

Russia, Caucasus.

#### Type material examined.

*Saprinus subvirescens*: Holotype: spec., “*subvirens* / Mén. Cauc (written) / Salian (red label, printed) / Holotypus (red label, printed) / round golden label” (ZIN).

*Saprinus foveisternus* Schmidt, 1884: Lectotype (present designation): female, glued on a mounting point with the following labels: “Baku” (written); followed by: “foveisternus / mihi typ.” (written); followed by: “Type” (brick-red label, printed); followed by: “coll. J. Schmidt” (printed); followed by: “foveisternus / Schmidt” (double-margined, written label); followed by: “Saprinus / foveisternus / Coll. Schmidt-Bickhardt” (printed); followed by: “Saprinus / foveisternus / Schmidt, 1884 / LECTOTYPE / des. Lackner 2014” (red label, written) (MNHUB). Paralectotype (present designation): female, with following labels: “Baku” (written); “Type” (brick-red label, printed); followed by: “foveisternus” (written); followed by: “Saprinus / foveisternus / Schmidt, 1884 / PARALECTOTYPE / des. Lackner 2014” (red label, written). Paralectotype (present designation): male, with the following labels: “Baku” (written); “Type” (brick-red label, printed); followed by: “Saprinus / foveisternus / Coll. Schmidt-Bickhardt” (printed); followed by: “Saprinus / foveisternus / Schmidt, 1884 / PARALECTOTYPE / des. Lackner 2014” (red label, written) (both MNHUB). Paralectotype (present designation), unsexed specimen, all tarsi, left meso- and metatibia missing, with the following labels: “Bakou / Caucase” (written); followed by: “in Col. Bonnaire” (written); followed by: “TYPE” (red label, printed); followed by: “coll. Dr. Auzat” (light green label, written); followed by: “foveisternus / mihi Typ” (written); followed by: “Saprinus / foveisternus / Schmidt, 1884 / PARALECTOTYPE / des. T. Lackner 2014” (red label, written) (MNHN).

*Saprinus syriacus* Marseul, 1855: Lectotype (present designation): male, with genitalia extracted, glued to the same mounting card as the specimen, right protibia broken off, glued next to specimen, right mid-leg and left hind leg missing, with the following labels: “90 / Saprinus / syriacus / Syrie m. ♂ / Laferté” (round yellow label, written); followed by: “Saprinus / syriacus m / Syria / 89” (yellow label, written); followed by: “Ti...further illegible text / 63” (tiny yellow label, written); followed by: “342” (orange label, written); followed by: “Schm. / 31” (written); followed by: “MUSEUM PARIS / COLL. / DE MARSEUL 1890” (printed); followed by: “TYPE” (red-printed label); followed by: “Sapr. subvires- / cens Men. / G. Dahlgren det” (printed-written); followed by: “Saprinus syriacus / Marseul, 1855 / LECTOTYPE 2014 / des. T. Lackner” (red label, written) (MNHN). This species has been described from unknown number of specimens and the lectotype designation fixes the identity of the species.

*Saprinus viridulus* Marseul, 1855: Lectotype (present designation): female, right metatarsus missing, with the following labels: small pink rectangular label, followed by: “89 / Saprinus / viridulus / Kurmaul / Deyr. Inde” (yellow, round label, written); followed by: “♀” (written); followed by: “MUSEUM PARIS / COLL. / DE MARSEUL 1890” (printed); followed by: “TYPE” (red-printed label); followed by: “Sapr. subnites- / cens Men. / G. Dahlgren det” (printed-written); followed by: “Saprinus viridulus / Marseul, 1855 / LECTOTYPE 2014 / des. T. Lackner” (red label, written) (MNHN). The species was described from unknown number of specimens and the lectotype designation fixes the identity of the species. Note that Dahlgren mistakenly identified it as *Saprinus subnitescens* Ménétriés (sic!). What he meant was *Saprinus subvirescens*, which was indeed described by Ménétriés, and not *Saprinus subnitescens*, which was in turn described by Bickhardt in 1909.

#### Additional material examined.

**Israel:** 2 ♂♂, Adullam, 17.v.2002, Y. Mandelik & V. Chikatunov lgt. (TLAN); 2 specs., Jerusalem, coll. Lange, no further data (MNHUB). **Tajikistan:** 5 ♂♂, Aruk Tau Mts., 20.iv.1978, A. Olexa lgt.; 1 ♂, Vachrobod, 8.vi.1966, A. Olexa leg.; 1 spec., Tigrovaya Balka, 2.–6.vi.1966, A. Olexa leg.; 1 ♂ Aruk-Tau (Garavuti), 29.iv.1978, M. Dvořák leg. (all exs. TLAN). 1 spec., Khujand, 21.iv.1921, Arkhangelskij leg.; 1 spec., Yagnob, Chichartob, 1892, Glasunov leg.; 4 specs., Pyanzh, from Khorod to Ishkashim, 6.vi.1928, Grishin leg.; 2 specs., Koktau Mts., near Kurgan-Tyube pass, 28.iv.1962, Guryeva leg.; 2 specs., Tian-Shan, Musart, vi.1894, Hauser leg.; 1 spec., Gandzhina, 15.iv.1966 (all exs. ZIN). 2 specs., Tian-Shan, Tekesthal, no further data (BMNH). 1 spec., Pyandzh Karatau ridge Mt. Astana 23.iv.1991, Gratchev leg.; 1 spec., Tigrovaya Balka reserve right side Vakhsh river 16.iv.1989, V. Gorbatovskiy leg. (all exs. CAS). **Turkmenistan:** 1 ♂, Turkmenistan, Firjuza, Ashghabad, 27.iv.1977, A. Olexa leg.; 1 spec., ibid, but 22.iv.1981, J. Strnad leg.; 7 ♂♂ & 1 ♀, Ashgabat, Nisa, 21.iv.1975, A. Olexa leg.; 1 ♂ Amurdarya-Kirki, 1.–5.v.1993, collector unknown; 1 ♂ & 1 spec. Kopet Dagh, near Firjuza, Vanovskij, 21.v.1991, Z. Kejval leg.; 1 ♂, Tekke, no further data; 1 ♂, Annau, Karakum, 21.iv.1981, A. Olexa leg.; 7 specs., Annau, 15.iv.1985, Kapler leg.; 1 spec., Firjuza, 18.iv.1988, Kafka leg.; 2 specs., Ashghabad, 14.iv.1988, S. Jákl leg.; 1 spec., ditto, but 29.iv.1991, R. Dunda leg.; 5 specs., Chuli, 12.-13.iv.1990, M. Kafka leg.; 4 specs., Tusly Kala, 11.iv.1990, R. Dunda leg. (all exs. TLAN). 1 spec., Kopet-Dagh Mts., further locality illegible, 15.x.1969, collector unknown, near *Opimus* burrow; 1 spec., Atrek River, Jacobson leg.; 1 spec., Chikishlyar, 30.–31.iv.1916, V. Ilin leg.; 2 specs., Kushka, 18.v.1936, Kreizberg leg.; 1 ♂ & 1 spec., Badkhyz, Penkhatchetpe, 6.iv.1971, Tikhomirova leg.; 2 ♂♂, Badkhyz, Kyzyl-Dzhar, 18.iv.1970, Tikhomirova leg.; 3 specs., Kelif, 18.iv.1988, Atamuradov leg.; 3 ♂♂ & 2 specs., Kara-Kala env., 19.v.1968, Tikhomirova leg.; 4 specs., Firjuza, 30.iii.1952, Kryzhanovskij leg.; 2 specs., Kopet-Dagh Mts., Geok-Tepe, Izgait, desert, 10.iv.1987, V.N. Prasopov leg.; 1 spec., Ashghabat, no further data; 3 specs., idem, but 23.iii.1903, G. Jacobson leg.; 7 specs., idem, but 19.iv.1929, sands, Vlasov leg.; 2 specs., idem, but 6.iv.1928; 7 specs., idem, but 6.vi.1925, Opanin leg.; 3 specs., idem, but 22.iii.1952, Romadina leg.; 8 specs., Repetek, 23.iii.1983, Krivoshatskij leg.; 1 spec., idem, but 17.iv.1914, Plavilstshikov leg.; 2 specs., idem, but 5.ii.1904, E. Fisher leg.; 1 spec., Germab, 12.x.1988, collector unknown; 2 specs., Murgab, no further data; 1 spec., Iolatanj, 1.iv.1927, Kizeritskij leg.; 4 specs., Tedzhen, 21.vi.1904, Arris leg.; 4 specs., Krasnovodsk (=Turkmenbashi), 28.iii.1919, B. Ilin leg.; 1 spec., Kopet-Dagh Mountains, 12 km S of Kyzyl-Arbat, 25.iv.1952, D. Stenberg leg.; 2 specs., Kara-Bogaz, 40 km N from Kyzyl-Arbat, 21.iv.1952, Sternberg leg.; 2 specs., 2–6 km N of Kara-Kala, 24.v.1952, Kryzhanovskij leg.; 1 spec., Sumbar river, 1894, Herz leg.; 1 spec., Firjuza env., 30.iii.1952, V. Ilichyov leg.; 1 spec., Annau, 12.v.1928, V. Gussakovskij leg.; 6 specs., Gyaurs, 3.iv.1984, Kh. Atamuradov leg.; 6 specs., Karabil, Shiram-Kuy, 22.iv.1984, Kh. Atamuradov leg. (all exs. ZIN). 1 spec., Amudaryinskiy reserve, Amudarya River, Nargiz island, 9–16.iv.1983, S. Alexeyev leg. (CAS). 1 spec., Badkhyz Penhancheshme, 6.iv.1971, Tikhomirova leg. (CND); 1 spec., Repetek, v. 1914, N. Plaviltshikov leg. (MNHN). **Jordan:** 1 ♀, Al Qatrana Saliya, 15.iv.2002, Wadi Mujib env., M. Snížek leg. (TLAN). **Kazakhstan:** 2 ♂♂ & 2 specs. Akkol, Jambul, 8.v.1979, A. Olexa leg.; 2 specs., ibid, but 10.v.1978, M. Dvořák leg.; 11 specs., Tjunja, Charyn River, 26.v.1994, collector unknown. (all exs. TLAN). 1 spec., Alma-Atinskaya oblast, Kuskuduk, 30.iv.1930, Kirschenbladt leg.; 1 spec., Alma-Atinskaya oblast, Karatalsk, 18.v.1930, Kirschenbladt leg.; 3 specs., SE Kazakhstan, Ilijskij, 22.viii.1911, Matissep leg.; 1 spec., Chelkar, 2.vi.1928, Olenev & Popov leg.; 1 spec., Aktyubinskaya oblast, Dzhilandy,11.vi.1908, D. Borodin & B. Uvarov leg.; 1 spec., Ostashkino, Almatinka, 20.vii.1928, Shnitnikov leg.; 2 specs., Mogyl Daumchar on River Emba, Temirsk region, 30.v.1908, Borodin leg.; 2 specs., Astau-Sardy, banks of River Emba, Temirsk region, 28.v.1908, Borodin leg.; 1 spec., Ak-Buta mountains, Temir, 2.vi.1908, Borodin leg.; 9 specs., Dzhilandy, Uralskaya oblast', Temirskij uezd, 11.vi.1908, D. Borodin & B. Uvarov leg.; 20 specs., surroundings of lake Inder, 4.vi.1907, B. Uvarov leg.; 1 spec., idem, but 10.vi.1907, A. Borodin leg.; 1 spec., Karatal (= Ush-Tobe), 16.v.1930, Kirschenbladt leg. (all exs. ZIN). 4 specs., North slope Talass ridge. near Talass vill., iv. 1993, no collector (CAS). 1 spec., Kapchagay env., Ili River, 10.v.1993, A. Ogarkov leg.; (CAS). **TURKEY:** 1 ♂ Cappadocia, 7.–10.vii. 1983, Avanos env., A. Olexa leg.; 1 ♂, Eskishehir, 5.v.1969, C. Holzschuch leg.; 1 ♂, Demircili, 70 km W Silifke, 5.iv.1992, O. Kapler leg. (all exs. TLAN). 1 spec., Kars env., Kaladzhika, 1.v.1915, Olsufyev leg. (ZIN); 1 spec., Anatolia, 29.iii.1977, 10 km SE Serefli Kochisar,Tuz Gölü, 1. Orient Exkursion, Inst. f. Zool. Mainz, Prof. R. Kinzelbach leg. (MNHN). **UZBEKISTAN:** 1 ♂ & 2 ♀♀ & 1 spec., Tashkent, 22.iv.1972, A. Olexa leg.; 3 ♂♂ & 2 specs., Samarkand, Aman Kutan, 21.iv.1972, A. Olexa leg.; 1 spec., ibid, but 20.iv.1972; 1 ♀, Khamsa-Abad, Ferghana, 26.iv.1972, A. Olexa leg.; 1 spec., Chimgan (Tian-Shan), 2500 m, 17.vii.1979, M. Dvořák leg.; 1 spec., Ak-Tash (Tashkent), 30.iv.1978, M. Dvořák leg.; 2 specs., 200 km W of Tashkent, Kyzyl-Kum Desert, Chardara (Koksu), 3.–5.v.1990, J. Turna leg. (all exs. TLAN). 3 specs., Ursatevskaya (=Khavast), 19.v.1920, I. Ivanov leg., on the ground in steppe; 1 spec., upper Upalanga river, Gissar Mt. range, 1898, Willberg, leg.; 1 spec., Tashkent, behind the Salar canal, 28.iii.1920, Ivanov leg.; 1 spec., Tashkent env., 16.v.1920, Ivanov leg.; 1 spec., idem, but 11.vi.1909, V. Grekov leg.; 1 spec., Sansar, 1892, Glasunov leg.; 4 specs., Kalma-Tai, 1892, Glasunov leg.; 2 specs., Tamdy, 1892, Glasunov leg.; 2 specs., Dzhizak, 1892, Glasunov leg.; 5 specs., Jgam-Berdy, 1892, Glasunov leg.; 1 spec., Kschtut Artutch, 1892, Glasunov leg.; 43 specs., Khodjent env., Golodnaya step, 23.iv.1903, G. Jakobson leg.; 1 spec., Bukhara region, Guzar-Tengi, Khoram, 28.iv.1897, Kaznakov leg.; 1 spec., Kammashi, N of Guzar, Bukhara region, 15.iv.1931, Gussakovskij leg.; 9 specs., Kizilcha, Bukhara region, Guzar env., 23.iv.1926, Gerasimov leg. (all exs. ZIN). **AZERBAIJAN:** 1 spec., Kobystan, Baku, 16.v.1975, A. Olexa leg. (TLAN). 1 spec., Kyurdamyr, near Baku, 15.v.1923, Bezrukov leg.; 3 specs., Pirsaat valley, 6.vii.1907, collector unknown; 1 spec., Baladzhary near Baku, 5.iv.1927, Kirschenbladt leg.; 2 specs., Lenkoran region, Nova Andreevka, 3.v.1923, Bezrukov leg.; 1 spec., Baku region, Belosovar, 5.v.1923, Bezrukov leg.; 1 spec., Ganja, no date, Dr. Kolenati leg.; 2 specs., Baku env., 18.iv.1927, Kirschenbladt leg. (all exs. ZIN). **KYRGYZSTAN:** 1 ♂, Kashka-su, v. 1984, J. Palička leg. (TLAN). 8 exs., Przhevalsk, 7.v.1930, Titov leg. (ZIN). 1 spec., Tian-Shan, Musart, no further data (BMNH). 1 spec., Kungey Alatau ridge, Grigoryevskoye canyon, 2000 m, 12–22.vii.1993 A. Ogarkov leg. (CAS).

**SYRIA:** 1 spec., Syria, no further data (BMNH). 1 spec., Palmyra, 10.–15.v.1995, P. Kabátek leg. (TLAN); 2 specs., Tadmor, Palmyra, Turkish Bath, 12.iii.1977, 1. Orient Exkursion, Inst. f. Zool. Mainz, Prof. R. Kinzelbach leg. (MNHN). **ARMENIA:** 1 spec., Rozdan, viii. 1981, Kletečka leg. (TLAN). **AFGHANISTAN:** 7 exs., Nengrahar prov., Jalalabad, 560 m, 20.iv.1967, D. Povolný et coll. leg. (MMBC). 1 ♂ & 1 spec., Laghman prov., Shamakat, 900 m, 22.iv.1972, Kabakov leg.; 1 spec., idem, but river Shamakat, ca 1000m, 31.iii.1972, Kabakov leg.; 1 spec., Herat prov., Anardara, 1200 m, 30.iii.1971, Kabakov leg.; 1 ♂ + 4 specs., Lataband pass, 30 km E Kabul, 4.iv.1970, Kabakov leg.; 1 spec., 15 km W of Jalalabad, 700 m, 30.iv.1972, Kabakov leg.; 1 spec., Nuristan prov., Petch, 1500 m, 21.x.1971, Kabakov leg.; 2 ♂♂ & 2 specs., Nuristan prov., Dara-i-Petch, 1600 m, 21.v.1971, Kabakov leg.; 14 specs., Kabul, 21.iii.1970, Kabakov leg.; 1 ♂ & 6 specs., idem, but 1800 m, 20.iii.1970; 4 specs., idem, but 26.iii.1971; 1 spec., idem, but 7.vi.1973; 1 spec., idem, but 15.iv.1970; 1 spec., idem, but 9.iv.1971; 1 spec., idem, but 12.iii.1971, 1800 m; 6 specs., idem, but 19.iii.1971 (all exs. ZIN).; 1 spec., 46 km NO Jalalabad, 800 m, Sar Kardou, 25.v.1962, Dr. K. Lindberg leg.; 21 specs., Ghourmatch, between Gaiar & Dala Morghab, carcass of *Herrison*, 16.iv.1959, Dr. K. Lindberg leg.; 6 specs., Decht-Bazar, 27.vii.1962, Dr. K. Lindberg leg., 1 ♀, prov. Bamyan, dirt track from Lanjaw to Bissoude, 2800 m, 23.viii.1978, G. Ledoux leg. (all exs. MNHN). **IRAQ:** 1 spec., Mesopotamia, without further data (ZIN). 1 spec., Euph. [=Euphrat?], no further data (BMNH); 5 specs., Mosul, no further data (MNHUB). **MONGOLIA:** 1 spec., Mongolia bor., without further data. (ZIN). **IRAN:** 1 ♂, Teheran, without further data; 1 spec., Kerman, Sargad, 4.v.1901, N. Zarudnij leg.; 1 spec., idem, but 13.iii.1928, B. Kuznetsov leg. (all exs. ZIN). 1 spec., Kerman, 4.iii.1935, H.E.J. Biggs leg. (BMNH). 1 ♂, Evine (Tehran), no date, Petrovitz leg. (CND). **GEORGIA:** Tbilisi, 19.iv.1880, collector unknown (ZIN). **RUSSIA:** 2 specs., Dagestan, Petrovsk, 1.v.1925, Kirishechenko leg.; 1 spec., Sarepta, Bekker leg., no further data; 1 spec., idem, but no date or collector; 3 specs., Stavropol reg., Faust leg.; 1 spec., Stavropol region, Roguli, 1925, collector unknown; 1 spec., Astrakhan, no date, A. Semenov-Tian-Shanskij leg.; 1 spec., Samara, Dr. Bols leg.; 1 spec., Stavropol'skij kray, Mitrofanovskoe, iv. 1925, collector unknown; 6 specs., Selitrennoe, Yenot uezd, 10.vi.1910, Chernovin leg. (all exs. ZIN). 1 spec., South Russia, Kalmykiya, 10 km S Tchernozemelskiy vill., 15.iv.1982, A. Zamesov leg.; 1 spec., Astrakhan reg. near Lower Baskunchak vill., Mt. Bogdo, 43°07.880'N, 46°49.168'E, 23–25.v.2013, A. Shadenkov leg. (all exs. CAS). **CYPRUS:** 6 specs., Cyprus, Nicosia, 19.iii.[19]35, Th. Shiakides leg. in cow dung (BMNH). **INDIA:** 1 spec., India, no further data (BMNH). 1 spec., Uttaranchal state, Naini Tal distr., near Sathkol vill., 20–28.vi.2006, S. Saluk leg.; (CAS). **PAKISTAN:** 1 spec., 22.ii.1978, Gujranwala, S. Kinelski leg. (CND).

#### Re-description.

Although this species has been recently re-described by the author (Lackner 2010: 205), and the reader is referred there for the exhaustive account of SEM micrographs and drawings of the mouthparts and sensory structures of the antenna, I prefer to repeat its re-description here for the sake of completeness of the revision, especially since the two subsequent species are morphologically rather similar and differ from the type species of the genus in their cuticular colour.

Body length: PEL: 2.25–3.00 mm; APW: 0.75–1.00 mm; PPW: 1.75–2.00 mm; EL: 1.50–1.90 mm; EW: 1.87–2.50 mm.

Body (Fig. 1) roundly oval, convex, cuticle pitch-black usually with greenish hue, shining, but older specimens can be completely dark without hue; legs, mouthparts and antennae dark brown; antennal club black.

**Figure 3. F3:**
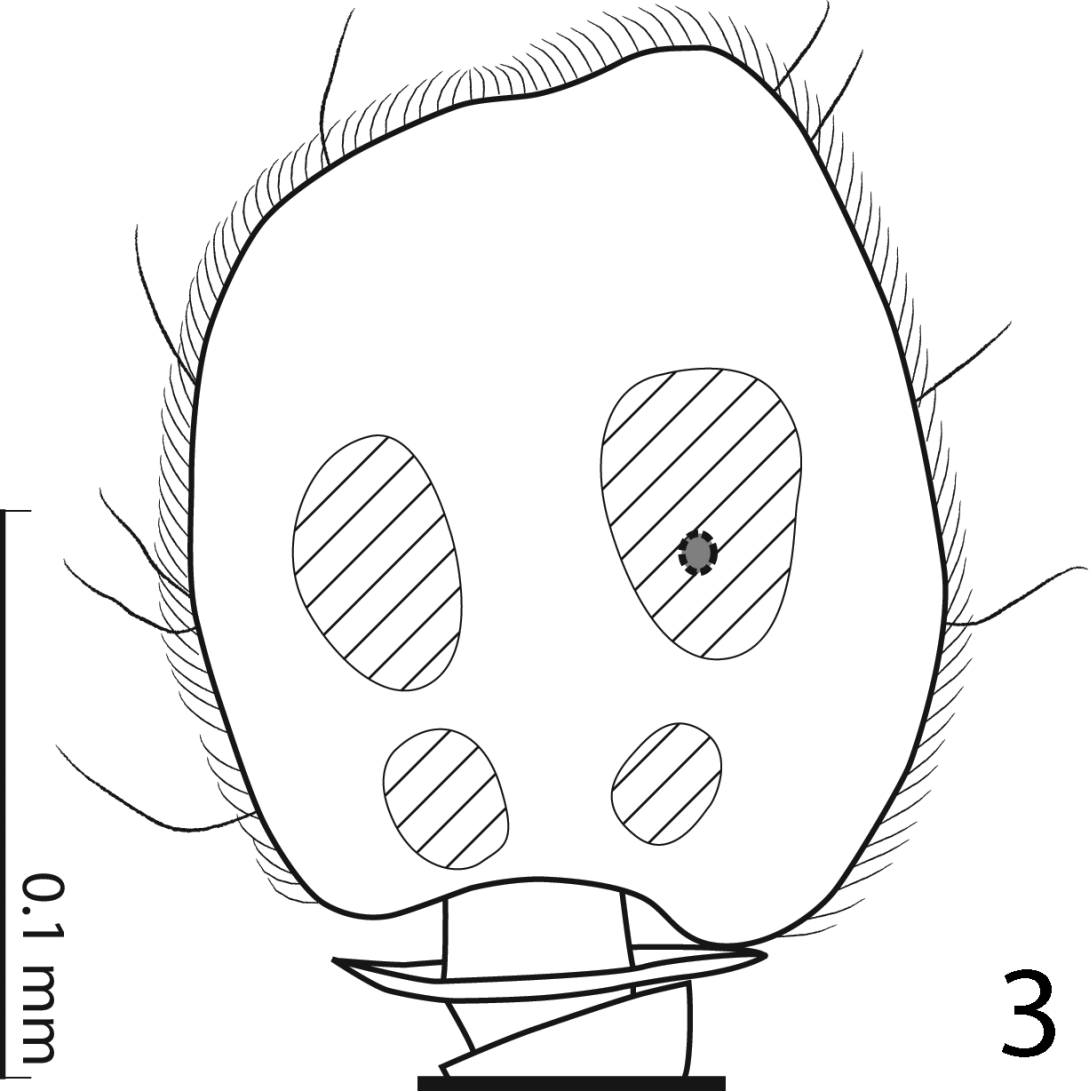
*Hemisaprinus subvirescens* (Ménétries, 1832) antennal club, showing the sensory structures.

Antennal scape (Fig. 2) not particularly thickened, with shallow sparse punctures and two short setae; club round, without visible articulation, entire surface with dense short sensilla intermingled with sparser longer erect sensilla; sensory structures of antennal club (Fig. 3) in form of four ovoid sensory areas on ventral side and one vesicle situated under internal distal margin.

Mouthparts: mandibles (Fig. 2) with rounded outer margin, laterally with deep dense punctures, moderately curved inwardly, mandibular apex pointed; sub-apical tooth obtuse, inconspicuous; labrum (for fig. see Lackner 2010, fig. 69) convex, densely punctate, anterior margin medially with a small convexity interrupting concavity; labral pits deep, each with two well-sclerotized long setae; terminal labial palpomere elongated, its width about one-third its length; mentum sub-trapezoid, anterior margin (for fig. see Lackner 2010, fig. 135) medially with deep notch surrounded with sparse short setae, lateral margins with single row of sparse shorter setae, several setae present also on disc of mentum; cardo of maxilla with few short setae; stipes triangular, with three short setae; terminal maxillary palpomere elongated, its width about one-fourth its length, approximately 2.5 times as long as penultimate.

Clypeus (Fig. 2) flat, constricted laterally, with coarse and dense punctures; frontal stria largely interrupted medially, for short distance prolonged onto clypeus, supraorbital stria well impressed, carinate; frontal disc (Fig. 2) with coarse and dense punctures; eyes convex, well visible from above.

Pronotal sides moderately (Fig. 1) narrowing anteriorly, apical angles obtuse, pronotal depressions vaguely impressed, almost absent, anterior incision for head shallow, almost straight in middle; marginal pronotal stria complete; pronotal disc laterally with longitudinal depression, with very coarse and dense punctures, punctures become finer and sparser medially; row of ovoid punctures present along pronotal base; pronotal hypomeron glabrous; scutellum small, but visible.

Elytral epipleuron with scattered fine punctures, area between marginal epipleural stria and elytral margin smooth; marginal epipleural stria fine, complete; marginal elytral stria straight, well impressed and slightly carinate, continued as weakened complete apical elytral stria; along marginal elytral stria a row of round dense punctures present. Humeral elytral stria weakly impressed on basal third; inner subhumeral stria present as short median fragment; all four dorsal elytral striae 1–4 weakly impressed, short, not reaching elytral half apically, in shallow punctures; fourth dorsal elytral stria basally vaguely connected with sutural elytral stria; sutural elytral stria well-impressed and complete, in deep punctures, apically connected with apical elytral stria; entire elytral disc with punctuation, punctures dense and coarse; along elytral margin, on elytral humeri and on interval between fourth dorsal and sutural elytral striae punctation weakens, extreme apex of elytra impunctate.

Propygidium and pygidium densely and coarsely punctate, punctures separated by about half their own diameter.

**Figure 4. F4:**
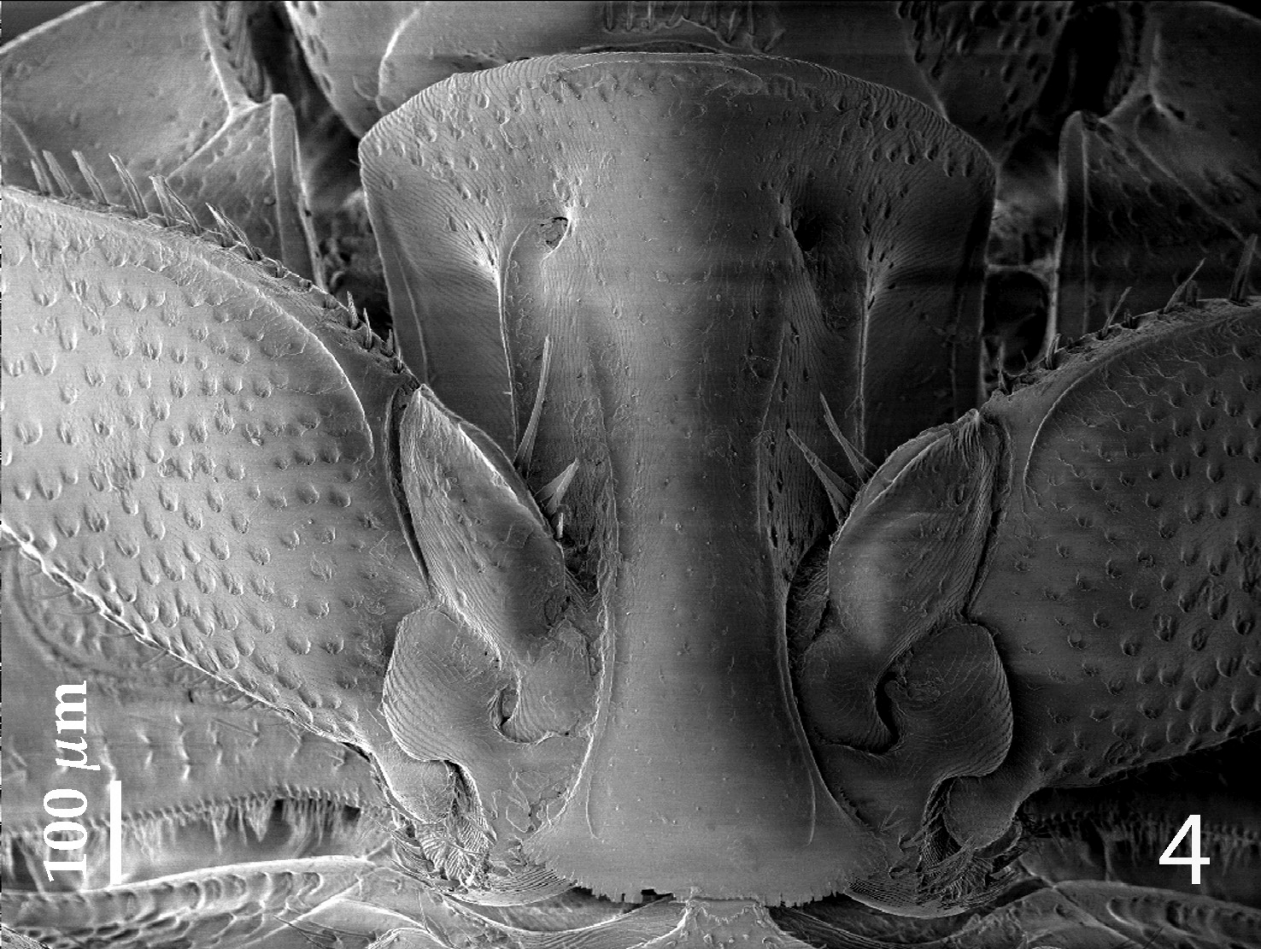
*Hemisaprinus subvirescens* (Ménétries, 1832) prosternum.

Anterior margin of median portion of prosternum (Fig. 4) almost straight; marginal prosternal stria present laterally and as a short anterior fragment; prosternal process concave, surface between carinal prosternal striae with scattered fine punctuation, laterally finely strigulate, punctures coarser and deeper; carinal prosternal striae well-impressed, on prosternal apophysis parallel, slightly divergent anteriorly, not connected apically; prosternal foveae deep; lateral prosternal striae carinate, sub-parallel, apically terminating in prosternal foveae.

Anterior margin of mesoventrite (for fig. see Lackner 2010, fig. 649) deeply emarginate medially; discal marginal mesoventral stria well impressed, carinate, slightly weakened medially; disc of mesoventrite with scattered punctuation; meso-metaventral sutural stria marked as straight row of coarse punctures; intercoxal disc of metaventrite (for fig. see Lackner 2010, fig. 649) flattened (in male with median longitudinal excavation), with fine punctures, becoming coarser and denser along posterior and lateral margins (especially behind hind coxa); lateral metaventral stria (for fig. see Lackner 2010, fig. 650) well impressed, carinate, almost straight, shortened; lateral disc of metaventrite (for fig. see Lackner 2010, fig. 650) slightly concave, with dense shallow setiferous punctures; metepisternum with even denser and coarser punctuation, punctures not setiferous; fused metepimeron with somewhat sparser punctures; metepisternum + fused metepimeron with metepisternal stria, interrupted on fusion between metepimeron and metepisternum.

Intercoxal disc of the first abdominal sternite laterally with incomplete stria; except for median part with coarse round punctures, becoming finer along posterior margin.

Protibia (for fig. see Lackner 2010, fig. 651) slightly dilated, outer margin with 5 moderately large triangular teeth topped with short rounded denticle, diminishing in size in proximal direction, followed by 4 tiny denticles; setae of outer row regular, rather short; protarsal groove deep, strigulate; anterior protibial stria complete apically; setae of intermedian row about as long as those of outer row, becoming more sclerotized apically; two tarsal denticles present near tarsal insertion; protibial spur short, bent, growing out from apical margin of protibia; apical margin of protibia posteriorly with 3 tiny denticles abutting each other; outer part of posterior surface (for fig. see Lackner 2010, fig. 651) obscurely variolate, separated from glabrous median part of posterior surface by vague boundary and row of short sclerotized setae; posterior protibial stria complete, with a row of tiny sclerotized setae becoming thicker apically; inner row of setae double, setae dense and short.

Mesotibia slender, outer margin with two rows of short denticles; setae of outer row regular, dense, shorter than denticles; setae of intermedian row shorter and finer than those of outer row, regular; posterior mesotibial stria almost complete; anterior surface of mesotibia (for fig. see Lackner 2010, fig. 645) strigulate-punctate; anterior mesotibial stria complete, terminating in single tiny inner anterior denticle; mesotibial spur short; apical margin of mesotibia anteriorly with two short denticles; claws of apical tarsomere slightly bent, shorter than half its length; metatibia slenderer and longer than mesotibia, in all aspects similar to it, but denticles on outer margin much sparser and claws of apical tarsomere slightly longer than half its length.

**Figures 5–12.**
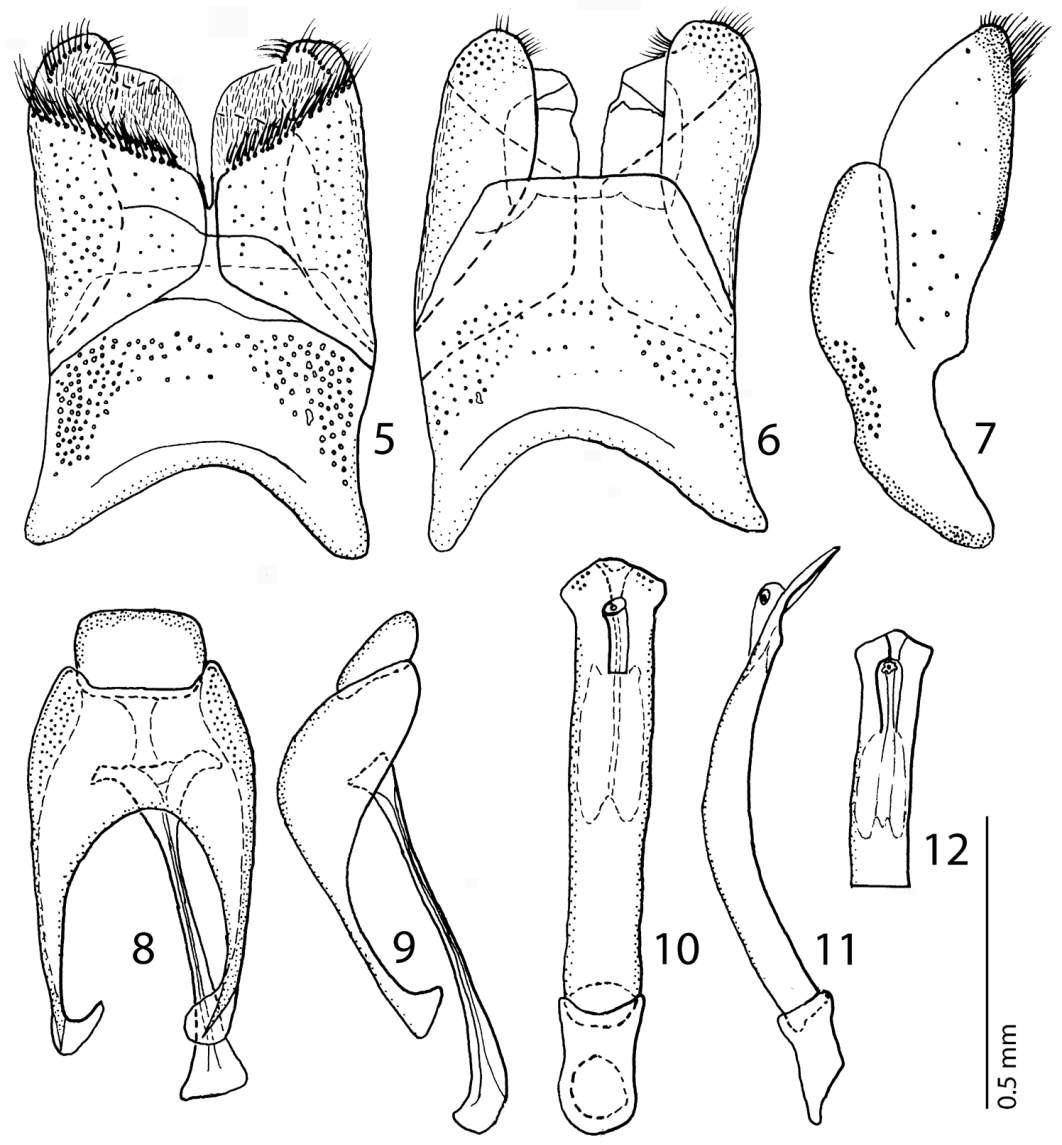
**5** *Hemisaprinus subvirescens* (Ménétries, 1832) 8^th^ sternite and tergite, ventral view **6** ditto, dorsal view **7** ditto, lateral view **8** *Hemisaprinus subvirescens* (Ménétries, 1832) 9^th^ + 10^th^ tergites, dorsal view; spiculum gastrale, ventral view **9** *Hemisaprinus subvirescens* (Ménétries, 1832) 9^th^ + 10^th^ tergites, spiculum gastrale, lateral view **10** *Hemisaprinus subvirescens* (Ménétries, 1832) aedeagus, dorsal view **11** ditto, lateral view **12** *Hemisaprinus subvirescens* (Ménétries, 1832) apex of aedeagus, dorsal view.

Male genitalia: Eighth sternite (Figs 5–6) widely separated medially, covered with pseudo-pores, apically with numerous close-set setae forming a conspicuous apical brush, velum with dense, much shorter and finer setae; on outer margin fringed with a single row of longer setae; eighth tergite (Fig. 6) apically straight; eighth tergite and eighth sternite fused laterally (Fig. 7). Ninth tergite (Figs 8–9) fused medially, laterally with pseudo-pores; spiculum gastrale (Fig. 8) almost parallel with apical end strongly, and basal end only slightly expanded. Aedeagus (Figs 10–12) slender; basal piece of aedeagus short, ratio of its length: length of parameres 1: 3.50; parameres fused almost along their apical three-fourths; aedeagus constricted apically, thence slightly dilated, curved ventrad (Fig. 11).

### Hemisaprinus lutshniki

Taxon classificationAnimaliaColeopteraHisteridae

(Reichardt, 1941)

**Figure 13.**
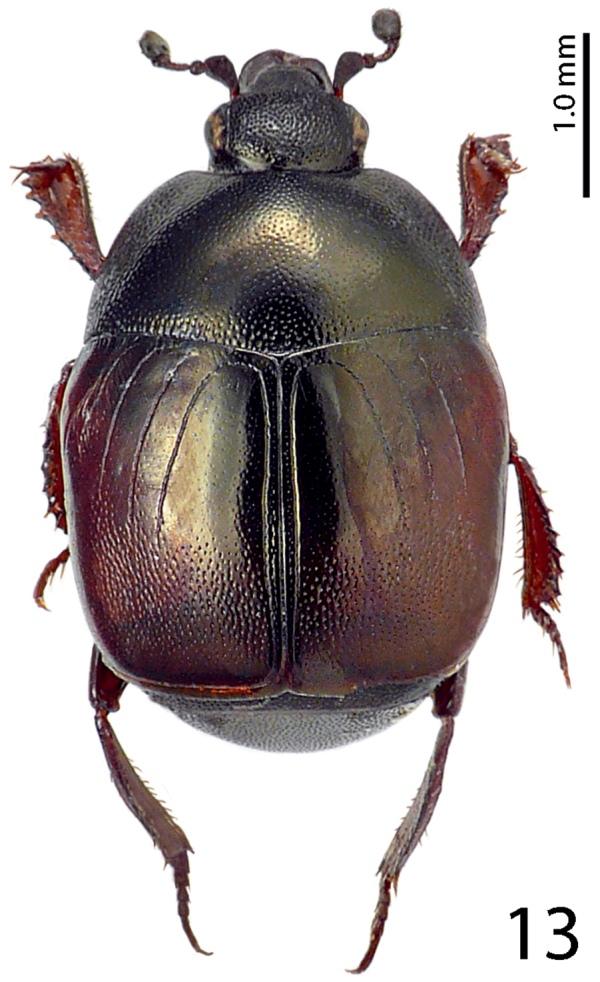
*Hemisaprinus lutshniki* (Reichardt, 1941) paratype, habitus.

Figs 13 [Fig F7] [Fig F8] [Fig F9] –25

Saprinus cribellatus ab. *lutshniki* Reichardt, 1941: 257, 392.
Saprinus (Hemisaprinus) lutshniki : Kryzhanovskij and Reichardt (1976): 183; Mazur (1984): 62; Mazur (1997): 231; Mazur (2004): 96.
Hemisaprinus lutshniki : Mazur (2011): 188.

#### Type locality.

Russia: Totskiy Rayon, near Samara.

#### Type material examined.

*Saprinus cribellatus* ab. *lutshniki*: Lectotype, sex unidentified, left mesotarsus missing, with following labels: circle, gold label, followed by: “Totskij lag / Samarsk. g. / 26.iv. 1917” (hand-written label in Russian); followed by: “*Saprinus cribell*. / a. *lutshniki* nov. / A. Reichardt det.” (printed-written); followed by: “Lectotypus / *Saprinus lutshniki* Rchdt. / Kryzhanovskij det., 66” (red label, printed-written). Paratypes: 1 ♂, with following labels: “Saratov / N.L. Sacharov” (black-margined label, written-printed); followed by: “Paratypus” (red label, printed). 1 spec., with the following labels: “O.B. Don 15.iv.[1]912 / Persianovka / B. Kizeritskij” (printed-written); followed by: “Paratypus” (red label, printed). 1 ♀, with following labels: “G. Temir Ural Obl. / 15.iv.[19]07 / D. Borodin & V. Uvarov” (printed-written in Russian); followed by: “Paratypus” (red label, printed); followed by: “Zoological / Institute RAS / St. Petersburg” (yellow label, printed); followed by: “lutshniki” (yellow, pencil-written label) and: “09-063” (yellow, pencil-written label) added by myself. 2 specs., with following labels: “Peremezhnoe, / okr. Uralska / Lyubishev 1.v.[19]33” (hand-written label in Russian); followed by red label, printed: “Paratypus” (all exs. ZIN).

#### Additional material examined.

**KAZAKHSTAN:** 1 ♂, River Ural near Kharkin, 14.v.1951, L. Arnoldi leg. (NCB); 56 exs., Ural River, near Kharkin, 14.v.1951, L. Arnoldi, under desert plants *Atraphaxys* (Polygonaceae) (ZIN); 1 spec., Ganibek nat. reserve, 49°23' N, 46°47' E, 1.v.2003, O. Khrulyova leg. (CAS); 2 specs., Ural River, Kharkin, 14.v.1951, L. Arnoldi leg. (MNHN). **RUSSIA:** 1 ♂, Orenburskaya oblast, 3 km NW Pervomaiskij, Donguz, steppe, 1.v.–28.vi.2009, Kozminykh V.O. leg. (TLAN). 1 spec., Volgograd, ovrag (=ravine) of the Tsaritsa River, 23.iv.1986, Matveev leg.; 2 specs., Samarskaya gubernia, Nikolaevskij distr., Bostanyhoglo leg., 1917; 1 spec., Samara, no date, Dr. Volz leg.; 1 spec., Kalmytskaya ASSR, Priozernij rayon, Tugtyn, 11.v.1976, Iviliev leg.; 1 spec., Petrovsk-port, N. Caucasus, 4.v.1931, M. Ryabov leg.; 1 spec., Tverskaya obl., Pokhot'-Krugloe, Zubtsovskij uezd, 30.v.1925, collector unknown; 3 exs., Kuybyshevskaya obl., Pestravskij rayon, kolkhoz “Rodina”, 14–15.v.1960, collector unknown; 4 specs., idem, but selo “Mosty”, 14.v.1960, under *Agropyron* plants in a ditch (all exs. ZIN); 1 spec., Kuybyshiev distr., 14.v.1960, Alejnikova leg. (BMNH); 1 spec., Astrakhan reg., 10 km S Upper Baskunchak vill., "Shikli" sands, 4.v.1995, I. Melnik leg. (CAS); 2 specs. Astrakhan reg., Palass distr., N side Elton lake, right side Khara River, 20–31.v.2006, A. Matalin leg. (CAS).

#### Re-description.

Body length: PEL= 2.75–3.35 mm, APW= 1.00–1.25 mm, PPW= 2.00–2.35 mm, EW= 2.25–2.60 mm, EL= 1.90–2.20 mm. Body (Fig. 13) rectangular oval, convex, elytra widest at humeri; cuticle of elytra on impunctate ‘mirror’ dark brown to black, on punctate part reddish-brown, shining, pronotum dark, almost black; body ventrally dark brown to almost black; abdominal ventrites (except for first visible) rufescent; legs, mouthparts and antennae rufo-castaneous; antennal club somewhat darker.

**Figure 14. F7:**
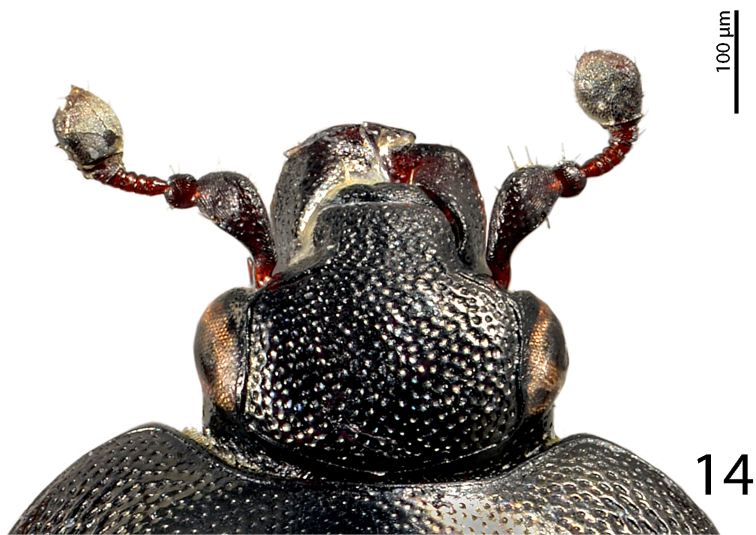
*Hemisaprinus lutshniki* (Reichardt, 1941) paratype, head, dorsal view.

**Figure 15. F8:**
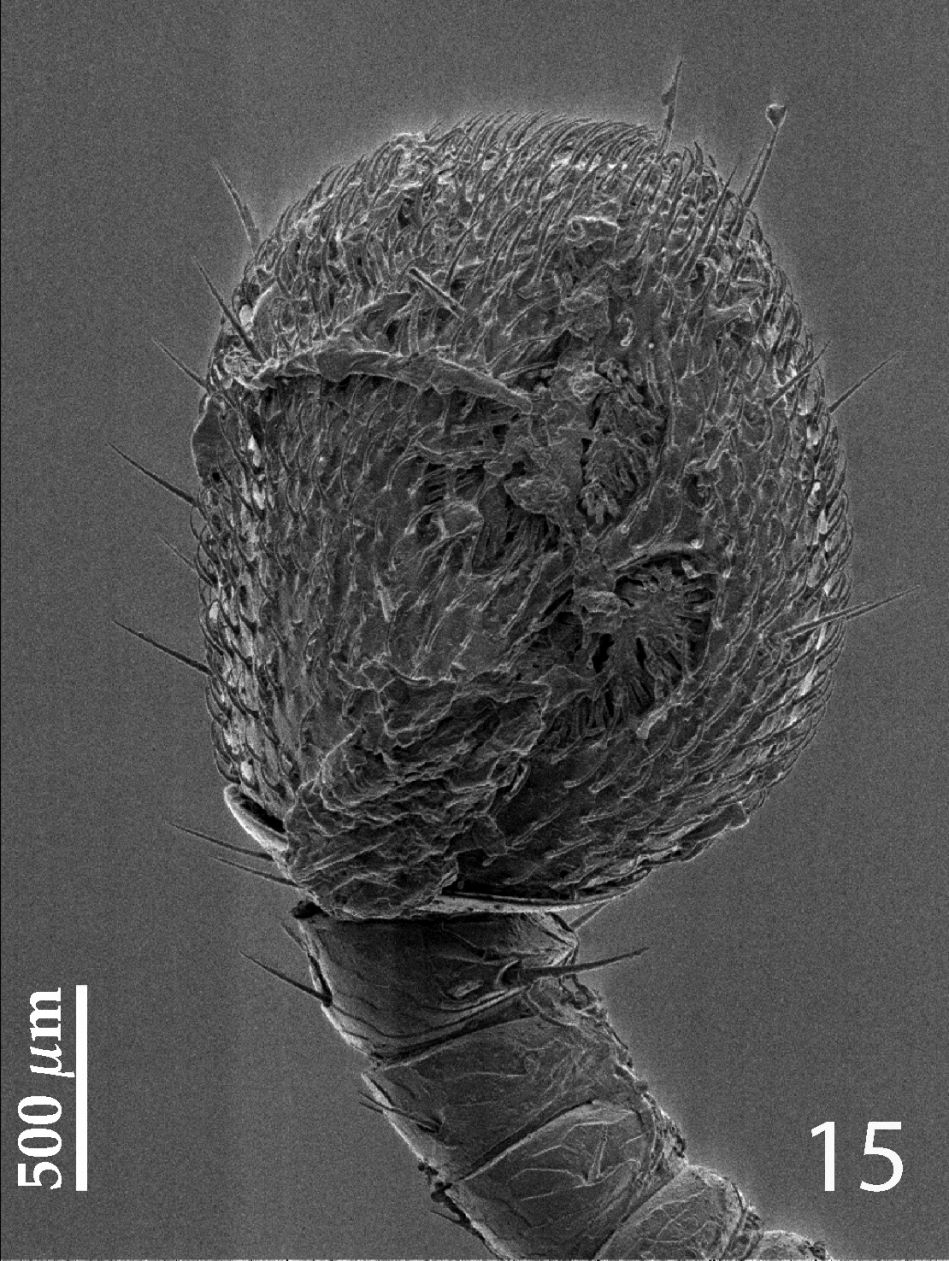
*Hemisaprinus lutshniki* (Reichardt, 1941) paratype, antennal club, ventro-lateral view showing sensory structures of the antenna.

Antennal scape (Fig. 14) slightly thickened, substrigulate, finely punctate, lower margin carinate, with few short setae; club (Figs 14, 15) round, pointed apically, without visible articulation, entire surface with dense short sensilla intermingled with sparser longer erect sensilla; sensory structures of antennal club in form of four ovoid sensory areas on ventral side (Fig. 15); vesicle(s) not examined.

Mouthparts: mandibles (Fig. 14) stout, densely punctate, mandibular apex pointed; sub-apical tooth of left mandible not examined; labrum convex, densely punctate, with slight median concavity interrupted by semi-globular convexity; labral pits deep, each with two well-sclerotized long setae; terminal labial palpomere elongated, about twice as long as pen-ultimate, its width about one-third its length; mentum sub-trapezoid, anterior margin medially with deep notch surrounded with sparse rather long setae, lateral margins with single row of sparse shorter ramose setae; cardo of maxilla with few short setae; stipes triangular, with three short setae; terminal maxillary palpomere elongated, pointed apically, about three times as long as pen-ultimate; its width about one-third its length.

Clypeus (Fig. 14) flat, gradually sloping down laterally, rugulose-lacunose; frontal stria broadly interrupted medially, for short distance prolonged onto clypeus, supraorbital stria well impressed, carinate; frontal disc (Fig. 14) very coarsely and densely punctate; eyes convex, well visible from above.

Pronotal sides (Fig. 13) on basal half moderately narrowing anteriorly, strongly narrowing on apical half; apical angles obtuse; median emargination for head shallow; pronotal depressions absent; marginal pronotal stria complete, somewhat weakened behind head; pronotal disc shining on most part, with sparse punctures separated by several times their diameter, laterally and behind head more coarse and dense punctures appear, punctures form a depressed band of confluent punctuation, between it and pronotal margin a narrow band with simple punctuation present; several rows of ovoid punctures present along pronotal base; pronotum with faint ante-scutellar depression; pronotal hypomeron asetose, in fine scattered punctures; scutellum well visible.

Elytral epipleura glabrous; marginal epipleural stria fine, complete; marginal elytral stria straight, well impressed and slightly carinate, continued as weakened complete apical elytral stria. Humeral elytral stria weakly impressed on basal fourth, doubled, surface between it and second dorsal elytral stria in longitudinal irregular strioles; inner subhumeral stria present as short median fragment; elytra with thin striae 1-4; striae with weak punctures within, except for first stria which is shorter than the others reaching approximately elytral half apically; fourth dorsal elytral stria basally connected with sutural elytral stria by broad arch; sutural elytral stria well-impressed and complete, fine punctures within, apically connected with apical elytral stria, between it and elytral suture a row of fine punctures present; elytral humeri and flanks almost impunctate, elytral disc along sutural elytral stria on apical two-fifths with dense, almost confluent punctation, forming longitudinal rugae; weakened punctuation slightly enters elytral intervals, apically punctuation weakens, leaving an impunctate band before extreme elytral apex; rest of elytral disk with large impunctate ‘mirror’, most prominent on 2-4 elytral intervals; this mirror occasionally bears fine scattered punctures, in most cases limited to second elytral interval.

Propygidium and pygidium densely and coarsely punctate, punctures separated by about half to their own diameter; interspaces with microsculpture.

**Figure 16. F9:**
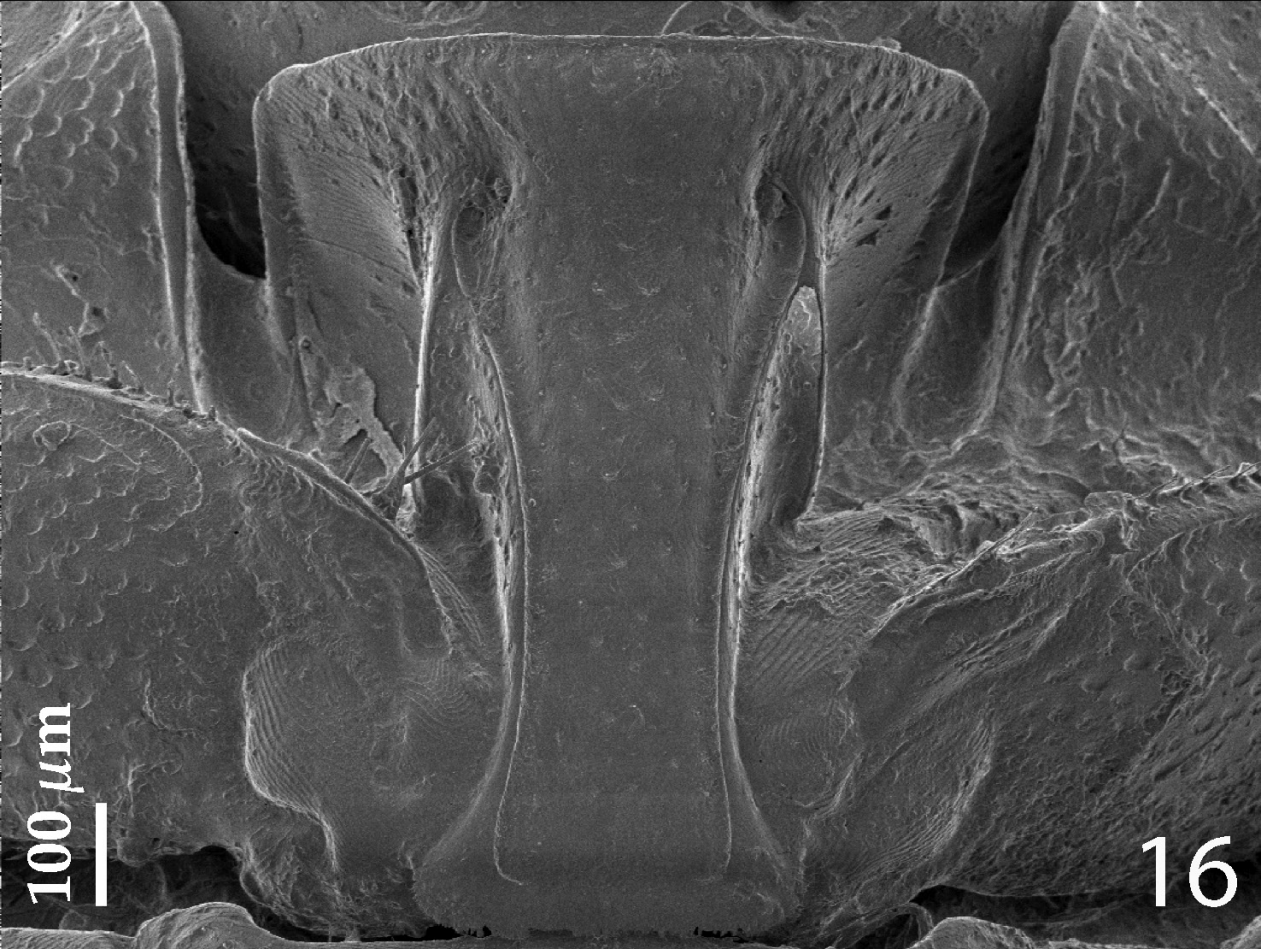
*Hemisaprinus lutshniki* (Reichardt, 1941) paratype, prosternum.

Anterior margin of median portion of prosternum (Fig. 16) rounded; marginal prosternal stria present laterally and as short anterior fragment; prosternal process on apical sixth distinctly elevated in respect to the remaining part; surface between carinal prosternal striae slightly convex, with scattered fine punctation, punctures surrounded by microsculpture; carinal prosternal striae well-impressed, parallel on prosternal apophysis, thence divergent anteriorly, terminating in deep and large prosternal foveae; lateral prosternal striae carinate, sub-parallel, apically terminating near the point where carinal prosternal striae enter prosternal foveae.

Anterior margin of mesoventrite broadly, but shallowly inwardly arcuate; discal marginal mesoventral stria well impressed, carinate; disc of mesoventrite with dense deep large punctures intermingled with much smaller microscopic punctuation; meso-metaventral sutural stria marked as straight row of punctures; intercoxal disc of metaventrite slightly convex with scattered microscopic punctures, becoming coarser and denser along basal margin; lateral metaventral stria well impressed, carinate, almost straight, shortened; lateral disc of metaventrite concave, with regular shallow large setigerous punctures; metepisternum with denser and coarser punctation, punctures almost confluent; fused metepimeron with somewhat sparser punctures; metepisternum + fused metepimeron with metepisternal stria.

Intercoxal disc of first abdominal ventrite incompletely striate laterally; on basal third with irregular scattered fine punctures separated by several times their own diameter; rest of first visible abdominal ventrite with scattered microscopic punctuation.

Protibia slightly dilated, outer margin apically with single low tooth topped by tiny denticle, in proximal direction three low triangular teeth topped by short rounded denticle appear, all three approximately of the same size, followed by another low tooth (occasionally bearing two tiny denticles), followed by a single tiny denticle growing out directly from outer margin of protibia; setae of outer row regular, rather short; protarsal groove rather deep; anterior protibial stria very shortened (absent?); setae of intermedian row situated on ridge delimiting proximal margin of protarsal groove; single tarsal denticle present near tarsal insertion; protibial spur short, bent, growing out from apical margin of protibia; apical margin of protibia posteriorly with three tiny denticles almost abutting each other; outer part of posterior surface obscurely variolate, punctate, separated from imbricate median part of posterior surface by vague boundary and row of short sclerotized setae; posterior protibial stria complete, bearing a row of fine sparse setae along its length, terminating in two tiny inner denticles; inner row of setae double, setae dense and short.

Mesotibia slender, outer margin with a single row of short denticles situated on low teeth; setae of outer row regular, sparse, about as long as denticles themselves; setae of intermedian row shorter and finer than those of outer row, regular; posterior mesotibial stria almost complete; anterior surface of mesotibia imbricate, with another row of approximately seven shorter denticles than those of outer row; anterior mesotibial stria complete, terminating in single tiny inner anterior denticle; mesotibial spur short; apical margin of mesotibia anteriorly with three short denticles; claws of apical tarsomere slightly bent, shorter than half its length; metatibia slenderer and longer than mesotibia, outer margin with approximately five short denticles situated on even lower teeth than those of mesotibia; apical-most tooth bearing two denticles; setae of outer row distinctly longer than denticles themselves; anterior face of metatibia punctate, with a row of approximately five tiny denticles; claws of apical-most metatarsomere longer than half of its length; metatibia otherwise similar to mesotibia.

**Figures 17–25.**
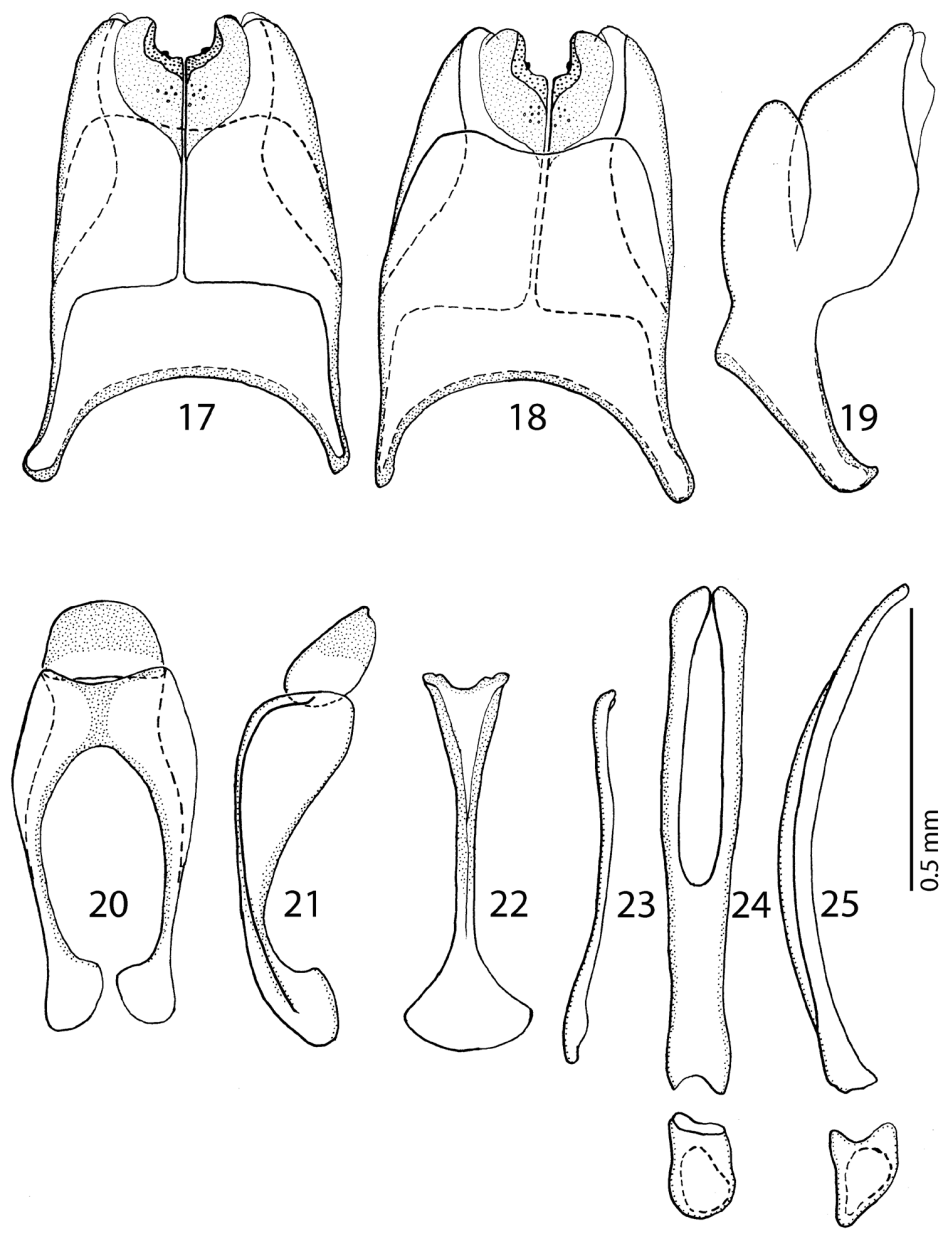
**17** *Hemisaprinus lutshniki* (Reichardt, 1941) paratype, 8^th^ sternite and tergite, ventral view **18** ditto, dorsal view **19** ditto, lateral view **20** *Hemisaprinus lutshniki* (Reichardt, 1941) paratype, 9^th^ + 10^th^ tergites, dorsal view **21** ditto, lateral view **22** *Hemisaprinus lutshniki* (Reichardt, 1941) paratype, spiculum gastrale, ventral view **23** ditto, lateral view **24** *Hemisaprinus lutshniki* (Reichardt, 1941) paratype, aedeagus, dorsal view **25** ditto, lateral view.

Male genitalia: Eighth sternite (Figs 17–18) longitudinally medially separated, apically with medially-sized velum covered with dense micro-pores and several larger pseudopores medially; eighth tergite inwardly arcuate; eighth tergite and sternite fused laterally (Fig. 19). Ninth tergite (Fig. 20) medially with strong longitudinal sclerotization, apically inwardly slightly arcuate; tenth tergite outwardly arcuate apically, basally slightly inwardly arcuate. Spiculum gastrale (Figs 22–23) basally strongly dilated, outwardly arcuate; apically slightly triangularly dilated, without typical inwardly-turned apical “tails”. Aedeagus (Figs 24–25) sub-parallel, parameres fused approximately on their apical halves, apex of aedeagus blunt. Basal piece of aedeagus rather short, ratio to parameres approximately 1:6; aedeagus curved laterally (Fig. 25).

#### Remarks.

This species is very similar to *Hemisaprinus cyprius*, differing from it chiefly by the presence of a second dorsal elytral stria, absent with *Hemisaprinus cyprius* and aciculate elytral punctuation, as well as shining pronotum (matt in *cyprius*).

### Hemisaprinus cyprius

Taxon classificationAnimaliaColeopteraHisteridae

(Dahlgren, 1981)

Figs 26 – 28

Saprinus cyprius Dahlgren, 1981: 112.
Saprinus (Hemisaprinus) cyprius : Mazur (1984): 62; Mazur (1997): 231; Mazur (2004): 96.
Hemisaprinus cyprius : Mazur (2011): 188.

#### Type locality.

Cyprus, Kyrenia.

#### Type material examined.

*Saprinus cyprius*: Holotype, ♀, side-mounted on triangular mounting point with left antennal club missing, female genitalia extracted, glued to another mounting label below the specimen, with the following labels: “Cypern, Kyrenia / 22/2 - 14/3 [19]62 / Th. Palm leg.” (printed); followed by: “HOLOTYPE / SAPRINUS / CYPRIUS / G. DAHLGREN / 25.1.1981” (written in black ink); followed by: “Zool. Mus. Lund Sweden / Type No. 2280: 1-2 / Histeridae” (printed-written); followed by: “**MZLU** / 2013 / 313” (green label, printed) (MZLU). Paratype, female, with following labels: “♀” (printed); followed by: “KYRENIA / CYPERN / 28.2.1962 / T. PALM LEG.” (written in black ink); followed by: “PARATYPE / SAPRINUS / CYPRIUS / G. DAHLGREN / 25.1.1981” (written in black ink); followed by: “Type No. / 2280:2” (printed-written); followed by: “**MZLU** / 2013 / 314” (green label, printed) (MZLU).

#### Re-description.

**Figure 26.**
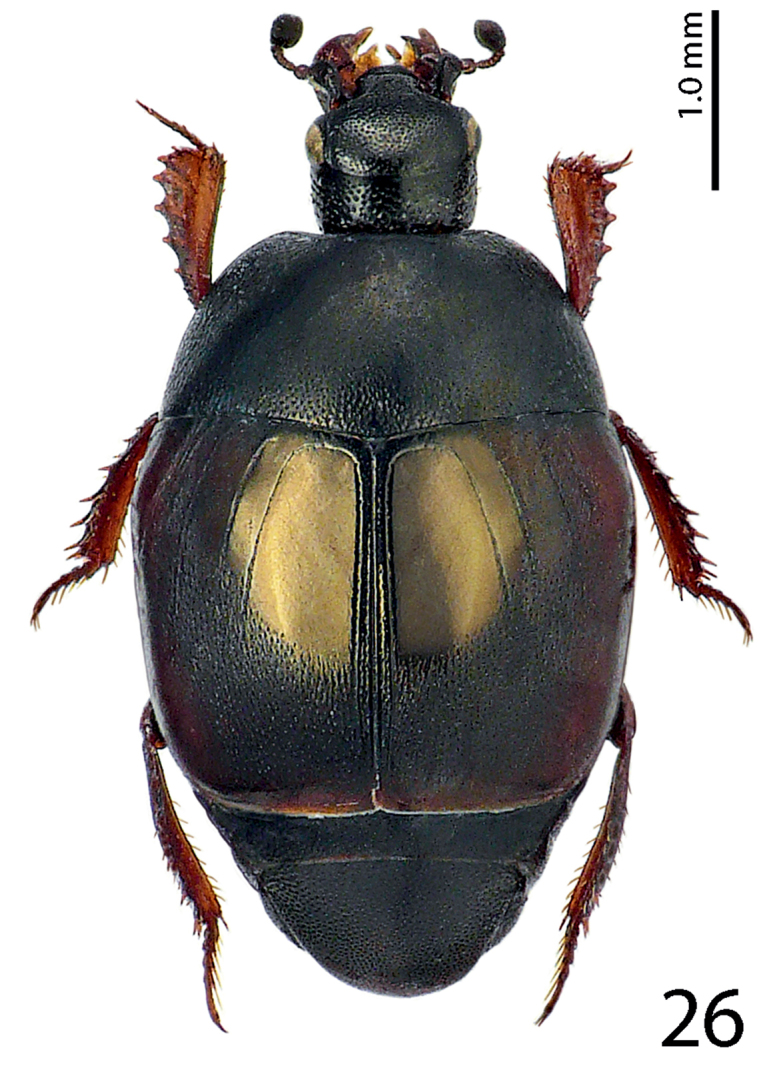
*Hemisaprinus cyprius* (Dahlgren, 1981) paratype, habitus.

Body length: PEL: 3.00–3.05 mm; APW: 1.00–1.05 mm; PPW: 2.15–2.25 mm; EL: 1.85–2.10 mm; EW: 2.35–2.50 mm. Body (Fig. 26) roundly oval, convex, elytra widest at humeri; cuticle of elytra castaneous, shining, pronotum dark, almost black, matt; body ventrally dark brown to almost black; abdominal ventrites (except for first visible) rufescent; legs, mouthparts and antennae rufo-castaneous; antennal club somewhat darker.

**Figure 27.**
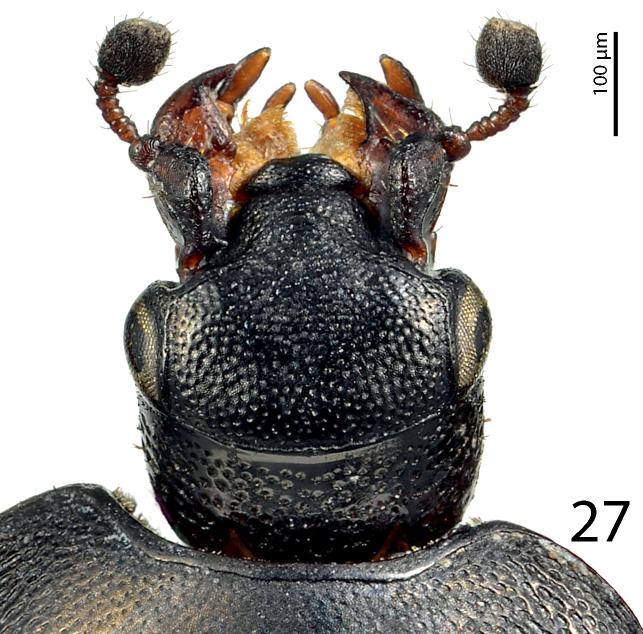
*Hemisaprinus cyprius* (Dahlgren, 1981) paratype, head, dorsal view.

Antennal scape (Fig. 27) slightly thickened, densely punctate, lower margin carinate, with few short setae; club round, pointed apically, without visible articulation, entire surface with dense short sensilla intermingled with sparser longer erect sensilla; sensory structures of antennal club in form of four ovoid sensory areas on ventral side; vesicle(s) not examined.

Mouthparts: mandibles with rounded outer margin, densely punctate, mandibular apex pointed; sub-apical tooth of left mandible not examined; labrum convex, densely punctate; labral pits deep, each with two well-sclerotized long setae; terminal labial palpomere elongated, about twice as long as pen-ultimate, its width about one-third its length; mentum sub-trapezoid, anterior margin medially with deep notch surrounded with sparse rather long setae, lateral margins with single row of sparse shorter ramose setae; cardo of maxilla with few short setae; stipes triangular, with three short setae; terminal maxillary palpomere elongated, pointed apically, about three times as long as pen-ultimate; its width about one-third its length.

Clypeus (Fig. 27) flat, gradually sloping down laterally, coarsely and densely punctate, punctures almost confluent; frontal stria largely interrupted medially, for short distance prolonged onto clypeus, supraorbital stria well impressed, carinate; frontal disc (Fig. 27) with coarse and dense punctures similar to those of clypeus, punctures in bottom with microsculpture; eyes convex, well visible from above.

Pronotal sides (Fig. 26) on basal half moderately narrowing anteriorly, strongly narrowing on apical half; apical angles obtuse; median emargination for head shallow; pronotal depressions absent; marginal pronotal stria complete, somewhat weakened behind head; pronotal disc matt due to very dense microsculpture, laterally with very coarse and dense punctures, separated by less than their own diameter, punctures become finer and sparser medially where they are separated by several times their diameter; several rows of ovoid punctures present along pronotal base; pronotum with ante-scutellar depression; pronotal hypomeron asetose, with fine scattered punctures; scutellum well visible.

Elytral epipleuron with scattered fine punctures; marginal epipleural stria fine, complete; marginal elytral stria straight, well impressed and slightly carinate, continued as weakened complete apical elytral stria. Humeral elytral stria weakly impressed on basal fourth, doubled, surface mesad from it with irregular longitudinal strioles; inner subhumeral stria present as short median fragment; elytra with thin, impunctate striae 1, 3-4 (stria 2 absent); striae stopping short of elytral half apically; fourth dorsal elytral stria basally connected with sutural elytral stria by broad arch; sutural elytral stria well-impressed and complete, fine punctures within, apically connected with apical elytral stria; elytral humeri and flanks almost impunctate, elytral disc along sutural elytral stria on apical 2/5 with fine regular punctuation, punctures aciculate, separated by about twice their own diameter, interspaces with very dense microsculpture, punctuation enters elytral intervals, reaching its climax along first dorsal elytral stria where it reaches elytral base, toward elytral apex microsculpture as well as punctuation weakens; extreme elytral apex impunctate.

Propygidium and pygidium densely and coarsely punctate, punctures separated by about half to their own diameter; interspaces with microsculpture.

**Figure 28.**
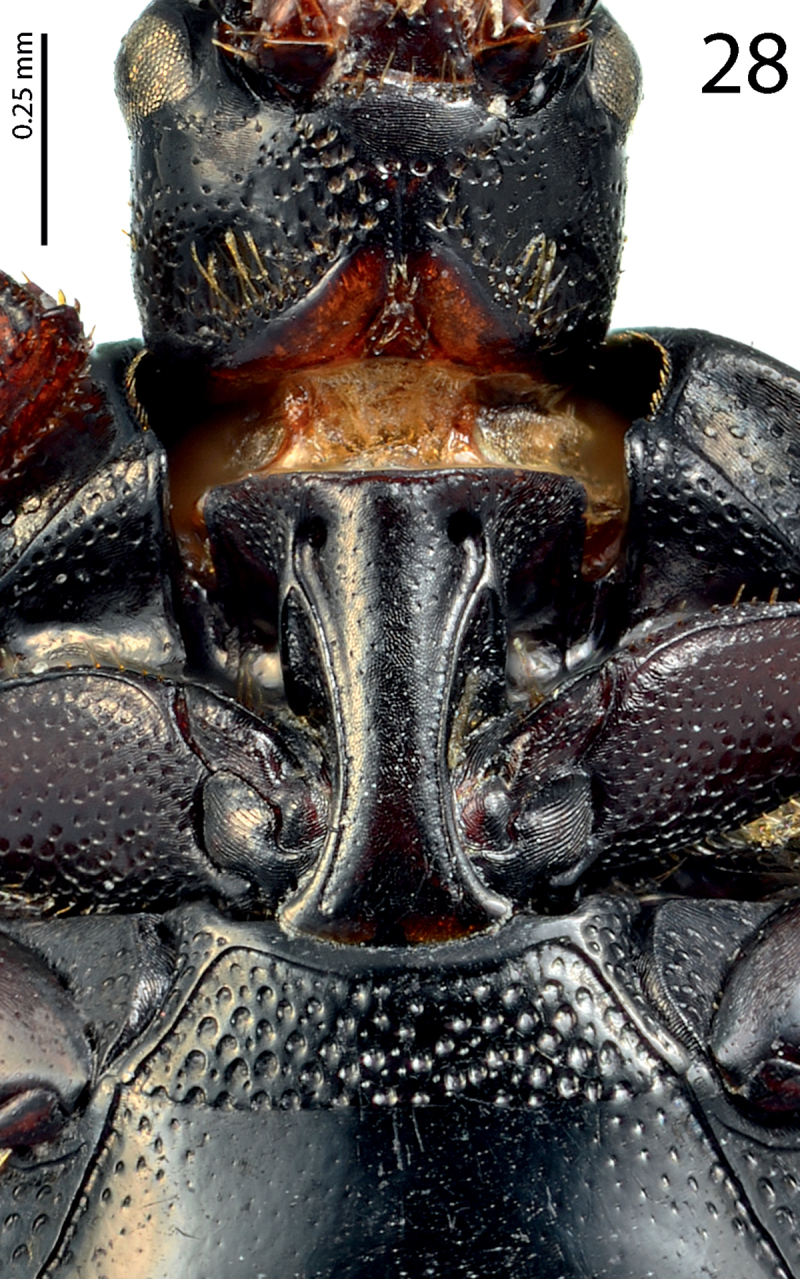
*Hemisaprinus cyprius* (Dahlgren, 1981) paratype, prosternum + mesoventrite.

Anterior margin of median portion of prosternum (Fig. 28) almost straight; marginal prosternal stria present laterally and as short anterior fragment; prosternal process between carinal prosternal striae slightly convex, surface between carinal prosternal striae with scattered fine punctuation, punctures surrounded by microsculpture; carinal prosternal striae well-impressed, parallel on prosternal apophysis, thence divergent anteriorly, terminating in deep and large prosternal foveae; lateral prosternal striae carinate, sub-parallel, apically terminating near the point where carinal prosternal striae enter prosternal foveae.

Anterior margin of mesoventrite (Fig. 28) broadly inwardly arcuate; discal marginal mesoventral stria well impressed, carinate; disc of mesoventrite with dense shallow large punctures intermingled with much smaller microscopic punctuation; meso-metaventral sutural stria marked as a straight row of punctures; intercoxal disc of metaventrite slightly convex with scattered microscopic punctures, becoming coarser and denser along basal margin; lateral metaventral stria well impressed, carinate, almost straight, shortened; lateral disc of metaventrite concave, with dense shallow large punctures; metepisternum with even denser and coarser punctation, punctures almost confluent; fused metepimeron with somewhat sparser punctures; metepisternum + fused metepimeron with metepisternal stria, which is almost unrecognizable under coarse punctuation.

Intercoxal disc of the first abdominal ventrite incompletely striate laterally; on basal third with irregular larger punctures separated by about their own to twice their diameter; rest of first visible abdominal ventrite with scattered microscopic punctuation.

Protibia slightly dilated, outer margin with four moderately large triangular teeth topped by short rounded denticle, diminishing in size in proximal direction, followed by three tiny denticles growing out directly from outer margin of protibia; setae of outer row regular, rather short; protarsal groove deep; anterior protibial stria shortened on basal half; setae of intermedian row not examined; two tarsal denticles present near tarsal insertion; protibial spur short, bent, growing out from apical margin of protibia; apical margin of protibia posteriorly with four tiny denticles almost abutting each other; outer part of posterior surface obscurely variolate, punctate, separated from glabrous median part of posterior surface by vague boundary and row of short sclerotized setae; posterior protibial stria complete, terminating in several tiny inner denticles; inner row of setae double, setae dense and short.

Mesotibia slender, outer margin with a single row of short denticles situated on low teeth; setae of outer row regular, sparse, longer than denticles; setae of intermedian row shorter and finer than those of outer row, regular; posterior mesotibial stria not examined; anterior surface of mesotibia glabrous, with another much sparser row of shorter denticles than those of outer row; anterior mesotibial stria complete, terminating in single tiny inner anterior denticle; mesotibial spur short; apical margin of mesotibia anteriorly with three short denticles; claws of apical tarsomere slightly bent, shorter than half its length; metatibia slenderer and longer than mesotibia, in all aspects similar to it, but denticles on outer margin much sparser, situated on even lower teeth than those of mesotibia; apical-most tooth bearing two denticles.

Male unavailable.

#### Remarks.

Dahlgren (1981) does not mention the absence of the second dorsal elytral stria, which is perhaps the best separating character from the similar species, esp. *Hemisaprinus lutshniki*.

##### Key to the species of the genus *Hemisaprinus*

**Table:** 

1(2)	Almost entirely dark-brown to black species, dorsal cuticle often with slight greenish metallic hue (Fig. 1) carinal prosternal striae stopping short of prosternal foveae, lateral prosternal striae terminate in them (Fig. 4); widely distributed species	*Hister subvirescens* (Ménétries, 1832)
2(1)	Usually bi-colored species: pronotum dark, almost black; elytra at least partly reddish-brown; dorsal cuticle without greenish hue, with slight to prominent bronze metallic tinge (Figs 13, 26); carinal prosternal striae terminate in prosternal foveae, lateral prosternal striae terminate near apices of carinal prosternal striae (Figs 16, 28).
3(4)	Second dorsal elytral stria absent; elytral ‘mirror’ impunctate, with bronze lustre; punctate part of the elytra with dense aciculate punctures and microsculpture; pronotum matt, medially almost impunctate (Fig. 26); species from Cyprus	*Hemisaprinus cyprius* (Dahlgren, 1981)
4(3)	Second dorsal elytral stria present; elytral ‘mirror’ with sparse scattered punctures, with slight bronze lustre; punctures on punctate part of elytra less dense, not aciculate, microsculpture absent; pronotum wholly punctate (Fig. 13); species from southern Russia, west Siberia and Kazakhstan	*Hemisaprinus lutshniki* (Reichardt, 1941)

## Discussion

Although Mazur (2011) did not provide any background information or justification for separating *Hemisaprinus* from *Saprinus* and erecting it as an independent genus he was motivated by the presence of the prosternal foveae in *Hemisaprinus* for his nomenclatural act (Mazur, pers. comm. 2014). Indeed, the presence of prosternal foveae is completely alien to *Saprinus* species and can justify the separation of *Hemisaprinus* from *Saprinus*. In the recently performed phylogenetic analysis aimed at disentangling the relationships of the genera and subgenera of the Saprininae (Lackner, unpublished) the type species of *Hemisaprinus*, *Hister subvirescens* was recovered deeply nested in the clade containing most of the type species of the Palaearctic and Nearctic taxa traditionally allied with *Saprinus* (sensu Mazur 2011). Its position is, however, not near the type species of *Saprinus*, *Saprinus semistriatus* and its placement in the clade was unambiguously supported by one synapomorphy: sensory structures of the antenna, which form regular patches on ventral side of the club and are usually four in number (Fig. 3). *Saprinus*, with 154 currently valid species is the most species-rich and widely distributed genus of the entire subfamily occurring on all continents except Antarctica (Mazur 2011). The genus *Saprinus* is most likely non-monophyletic and its phylogeny-based revision is highly necessary (see also Lackner 2010).

*Hemisaprinus*, although presumably related to *Saprinus* based on external as well as genitalic characters (Lackner, unpublished), is presumed to be monophyletic sharing the synapomorphy of present prosternal foveae. It contains three species that, on one hand, share the synapomorphy of the presence of prosternal foveae, on the other hand, however, the species differ in the arrangements of the two sets of prosternal striae. Carinal prosternal striae of *Hister subvirescens* do not enter the prosternal foveae; while the lateral prosternal striae do. In the case of the two other species (*Hemisaprinus lutshniki* and *Hemisaprinus cyprius*) the carinal prosternal striae do terminate in the prosternal foveae, while the lateral prosternal striae terminate near the apices of carinal prosternal striae. According to my recent studies on the morphology of the Saprininae, the configuration of the two sets of prosternal striae was found to be a rather variable character, even within one genus (and even within one species!) and I was unable to score this character unambiguously or parse it into discrete character states. Hence, I refrained from using this character in my phylogenetic studies (Lackner, unpublished) and do not use the different arrangements of the two sets of striae to further split *Hemisaprinus*.

On the other hand, a very similarly structured prosternal process, including the prosternal foveae is found among some members of the Nearctic and Neotropical subgenus *Hesperosaprinus* Wenzel, 1962 of the genus *Euspilotus* Lewis, 1907. The author is not familiar with most members of this species-rich subgenus (45 currently valid species, Mazur 2011), but based on the morphology studied and dissections of the antennal club of the type species of the subgenus, *Euspilotus (Hesperosaprinus) assimilis* (Paykull, 1811) at least two fundamental differences among this species on one hand, and members of *Hemisaprinus* on the other hand, were observed. The prosternal foveae of *Euspilotus (Hesperosaprinus) assimilis* are connected by marginal prosternal stria, whereas such stria is lacking in members of *Hemisaprinus*; and, furthermore, the sensory structures of the antenna of *Euspilotus (Hesperosaprinus) assimilis* consist of two (ventral and dorsal) circular sensory areas and a single, ball-shaped vesicle. The antennal character perhaps best separates the members of the two respective genera *Hemisaprinus* and *Euspilotus*. However, further studies of this enigmatic structure are required, especially among Nearctic and Neotropical Saprininae.

Dahlgren (1981) had some doubts about the placement of *Saprinus cyprius* into the subgenus *Hemisaprinus*, and remarked that: “Because the prosternal foveae are normally present in [*Saprinus (Hemisaprinus)*] *subvirescens* and [*Saprinus (Hemisaprinus)*] *lutshniki* this species [*Saprinus cyprius*] should be assigned to the subgenus *Hemisaprinus*. However, the appearance of *cyprius* is very different from these [two] species, and thereby the subgenus would be very heterogeneous. It seems that the genus *Saprinus* shows a tendency to produce species with prosternal pits and this tendency becomes manifested in different branches of the genealogical tree”. Although Dahlgren (1981) did not explicitly place *Saprinus cyprius* into *Hemisaprinus*, Mazur included it in this subgenus already in the first edition of his catalogue (1984) without providing any reason. Presumably it was likewise the presence of the prosternal foveae that inspired this placement.

## Supplementary Material

XML Treatment for Hemisaprinus

XML Treatment for Hemisaprinus subvirescens

XML Treatment for Hemisaprinus lutshniki

XML Treatment for Hemisaprinus cyprius
